# Towards painless and productive research relationships: reflections on study design by a researcher with chronic pain for participants with chronic pain

**DOI:** 10.3389/fpain.2024.1450667

**Published:** 2025-02-05

**Authors:** Catherine Wilkinson

**Affiliations:** School of Education, Liverpool John Moores University, Liverpool, United Kingdom

**Keywords:** ankylosing spondylitis, Crohn's disease, ethics, pain, participant, research relationships, researcher

## Abstract

Building flexibility into the research design of a study allows for responsiveness to the embodied and fluctuating nature of participants’ chronic illnesses, which may be shaped, for instance, by flare-ups and periods of remission of acute pain. Whilst the methodology literature has, to some extent, considered how to accommodate the pain of research participants when designing a study, consideration of how methodological choices are responsive to the researcher's pain needs has not to date been foregrounded. From the perspective of a researcher with Ankylosing Spondylitis (AS), a form of inflammatory arthritis characterized by chronic pain, and Crohn's disease, a type of inflammatory bowel disease, characterized by stomach and joint pain, this paper provides insight into pain and researcher-participant relationships, from the perspective of a researcher in pain, designing a study to accommodate her own pain needs, as well as anticipating the needs of prospective participants in pain. This paper proposes the use of flexible, remote, and asynchronous research methods as ways to make studies inclusive for researchers living with pain, whilst fostering the most fruitful research relationships with participants who also live with pain, thereby moving towards a position of shared vulnerability. It also highlights the relative absence of the researcher's needs and possible vulnerability in ethics forms and considered by research ethics committees, in comparison to the needs and vulnerability of participants.

## 1 Introduction

In academic literature considering how to design and undertake research studies in order to produce the most fruitful researcher-participant relationship, it is the participants' needs that are often articulated and catered to. For instance, ensuring the participants feel comfortable in a location where interviews are to be conducted (1), and accommodating participants regarding timings of study visits and interview lengths (2). But there has been very limited consideration of, or at least articulation of in published materials, designing research studies to meet the researcher's needs which, as I argue with this paper, are also important. This lack of acknowledgement in published literature also maps onto institutional ethics forms which are heavily concerned with a proposed study being appropriate (i.e., non-burdensome) for research participants, with many questions asked to ensure the study design is ethically sound, in line with the participant's assumed vulnerability (3), yet with limited questioning to determine the appropriateness of the study design from the researcher's perspective. Further, whilst institutional ethics may be concerned with protecting the researcher from harm that may come with lone working when undertaking interviews (4), or from distress caused by an emotional research topic (5), there is little to no consideration of the researcher's physical health needs and how a study has been designed to meet these needs, which may be particularly important for researchers with a chronic illness or disability.

This paper is written from the perspective of a researcher living with two chronic illnesses: AS, a form of inflammatory arthritis characterized by chronic pain, and Crohn's disease, a type of Inflammatory Bowel Disease (IBD), characterized by stomach and joint pain. It provides insight into pain and researcher-participant relationships from the perspective of a researcher in pain, designing a study “IBD, School and Me: An Exploration of the Emotional, Embodied and Affective Experiences of Everyday School Life for Children with Crohn's Disease and Ulcerative Colitis” to accommodate my own pain needs, as well as anticipating the needs of prospective participants in pain. This paper has two key premises. It proposes the use of flexible, remote, and asynchronous research (an approach in which the respondent records their response on their own time - within a given time frame) as a way to make studies inclusive for researchers living with pain, whilst fostering the most fruitful research relationships with participants who also live with pain. It also highlights the relative absence of the researcher's needs and possible vulnerability in ethics forms and considered by research ethics committees, in comparison to the needs and vulnerability of participants.

This paper is structured as follows. I will first provide insight into the nature and context of the proposed study, and will also outline my positionality in relation to this study. Then, I provide an insight into key debates concerning research relationships and the different framings of researcher-participant relationships, before moving on to review studies concerned with researching when disabled or chronically ill. The paper then explores the decisions I made surrounding the research design and methods of data collection in the study referred to herein as “IBD, School and Me” to accommodate both my own pain, and the anticipated pain needs of prospective research participants, in order to foster the most productive research relationships. Moving towards a position of shared vulnerability, this paper concludes by calling for a centering of the researcher, alongside the participant, both in study design and research ethics.

### 1.1 The nature and context of the proposed study

The aim of the research study on which this paper is based “IBD, School and Me” is to provide insight into the emotional, embodied and affective experiences of everyday school life for children with IBD, specifically Crohn's disease and ulcerative colitis. This study will seek to recruit up to 20 school children and young people (aged 5–16 years) with IBD as participants.

The following research questions were developed to guide the study:

1. How do children and young people with IBD use and experience spaces at school?
2. How do children and young people with IBD manage their bodies and identities in the school setting?
3. In what ways are creative, cathartic and care-full methods beneficial in exploring everyday school life for children and young people with IBD?

IBD can cause severe stomach pain, an urgent need to use the toilet, diarrhea (with or without bleeding), joint pain, extreme tiredness, nausea, and loss of appetite. Some children with IBD may also have delayed growth, weight loss, eye problems, mouth ulcers and anemia (6). IBD symptoms can fluctuate between periods of remission and acute flare-ups (7). Owing to the range of symptoms experienced, the school environment poses some significant challenges to children with IBD, including but not limited to: urgency to use the toilet, anxiety around eating school dinners or packed lunches, taking medication amongst peers, and long school days and the associated fatigue (8). Whilst important, existing research into IBD and school has not focused on the emotional, embodied and affective experiences of IBD in this setting. This is an important neglect that the proposed study aims to address, because improving children and young people's relationships with space, place and their bodies in the school setting has the potential to improve their attendance and thus their academic achievement long-term.

### 1.2 Positionality

I have AS, an autoimmune condition and type of inflammatory arthritis characterized by chronic pain. Whilst the symptoms of AS can vary from person to person, I experience severe spinal pain (including neck pain), lower back pain and stiffness, and also peripheral joint pain, including hip, knee, and elbow. With the peripheral joint pain, I experience pain and swelling caused by inflammation where a tendon joins a bone. Accompanying the pain is also fatigue. It took four years of persistent doctors’ appointments, physiotherapy, and Accident and Emergency hospital visits before I was finally diagnosed with AS following a human leukocyte antigen B27 blood test and magnetic resonance imaging scan in 2019. I also have Crohn's disease, sharing this health condition with some of the potential research participants in the proposed study. I was diagnosed in 2023 yet had experienced symptoms (frequent loose stools, weight loss, abdominal pain and fatigue) for around a year prior to diagnosis, and was diagnosed following two colonoscopies, which found a stricture in the terminal ileum. As well as the key bowel symptoms, I also experience pain in the joints in my back, hands, feet, arms and legs, as well as stomach pain. Whilst I am currently receiving biologic treatment which helps to control some of the everyday symptoms for both my AS and Crohn's, I still experience frequent painful flare-ups. Since I draw on my own personal situation, first person has been used in instances throughout this paper to allow the centering of myself as the researcher, something I claim is necessary in wider research practice.

## 2 Research relationships

Research of any variety pulls the researcher into relationships; these relationships shape the setting in which emotions are expressed or suppressed (9). A researcher holds various relationships with multiple groups, including their institution and their research participants (10). The researcher-participant relationship, the focus of this paper, has been conceptualized in numerous ways, by different scholars across diverse disciplines. One recurring debate is centered on power relations, with many researchers acknowledging the asymmetry of power between participant and researcher, where the researcher is often thought to be dominant^1^ and the participant positioned as vulnerable (10). Many studies have discussed attempts by researchers to minimize the power differentials; for instance, through the way researchers dress when undertaking interviews (12), through the settings they choose to undertake their interviews in, particularly in research with children where certain spaces can be skewed towards adult power and authority (1), and through the manner in which they pose interview questions, for instance asking questions in a friendly conversational tone, promoting a two-way exchange, as opposed to a stringent question and answer structure (13).

Writing about producing knowledge with care, Sander (14) discusses the importance of building mutually caring researcher-participant relationships, with specific reference to Gilligan's (15) ethics of care theory. According to Gilligan (15), there are two ways of thinking ethically. The first connects to the ethics of justice and rights and emphasizes what is right, good and just. The second relies on the ethics of care and focuses on maintaining healthy relationships and on questions of what is needed, when, where and by whom to do so. Butcher (16) likewise discusses emotionally engaged approaches when researching with vulnerable participants, taking into consideration the researcher's positionality, personal experience and proximity to the field. These studies do not, however, consider how relationships with participants may be developed from the choice of methods a researcher selects at the point of designing their study. Certain research, namely fieldwork (17), ethnographic research (18) and participatory research (19) involves prolonged and often personal interaction between the researcher and participants, and in research of this nature, more intimate relationships are more likely to be formed. Much literature has focused on how to foster the most productive research relationships, recognizing that research relationships can be friendly, professional, or somewhere in between. For instance, some argue that intimacy between researcher and participants can lead to long-term genuine friendships, whilst other researchers have concluded they were “not, and could not, be friends” with their participants [see Wilkinson and Wilkinson (12), p. 4, see also Blackman (20)]. Sometimes the line between participant and friend becomes blurred, causing the researcher to step back and reflect on the nature of this relationship (21), and even to take stock of whether a participant is indeed telling them a piece of information as a researcher or a friend [see Wilkinson (22)]. Kraft et al. (23) discuss bridging the researcher-participant gap to build effective research relationships, with a focus on processes of introducing a study to potential participants and gaining their consent. However, whilst important, this commentary does not consider the decisions made by the research team that precede the design of study materials, for instance concerning research design, and how these may help to bridge the researcher-participant gap.

### 2.1 Researching when disabled/chronically ill

So often, the default assumption is that disabled or chronically ill people will only be involved in research as participants, or worse still, as disempowered subjects. There often appears to be a significant gap in the relationship between the omnipotent “well” researcher and the vulnerable “unwell” participant. There is important work underway as part of the Disability Matters study (24). Asking “What kinds of research methodologies represent disabled people and their health priorities?” this project will produce a critical interdisciplinary literature review assessing the use of research methodologies undertaken previously on disability and health. Findings from this review will feed into online methodology workshops undertaken with disabled researchers. Until this literature review is published, it is difficult to locate work whereby researchers centre their own illnesses and disabilities when undertaking empirical research.

Some work located includes Ciotti's (25) autoethnographic research. Ciotti (25) utilizes reflexivity to explore the experience of Lyme disease while holding co-occurring identities as a health professional, mother, and researcher investigating the embodied experience of being a Lyme disease patient. The author moves towards a position of shared vulnerability with her participants, disclosing that she too has a chronic illness. Ciotti (25) reflects on how her insider membership may result in greater candor between some research participants and herself as the researcher, leading to richer data collection. Ciotti (25) encourages other health researchers to engage in ongoing reflexive practice, recognizing the value that her perspective as both researcher and patient offers to experiences of chronic illness. Whilst only a temporary condition, discussing researching with a broken arm, Ho (26, p. 78) articulates “finding sisterhood” with participants in her study. For Ho (26), her broken arm highlighted her vulnerability as a researcher and opened doorways to navigate different research methods, documentation and presentation of lived experiences. Ho (26, p. 80) believes her broken arm allowed for “identification through injury”, and served as an invitation for participants to share their vulnerabilities in their everyday lives. Methodologically, she considers autobiography through documentary films as one way of validating suffering and aestheticizing pain through the sharing of experiences.

Existing work on go-along interviews (interviews where the researcher accompanies a participant on the move through the environment) has reflected on the challenges of undertaking this method of data collection as a disabled researcher (27–29). Most recently, Larrington-Spencer et al. (29, p. 1) discuss go-along interviews as “emotionally, cognitively and physically demanding”. The authors emphasize the importance of care in go-along interviews, noting that these interviews can be both physically and cognitively tiring. They argue that care has been largely neglected in previous research on this method, particularly the relational aspects of care, such as the well-being of the researcher. Importantly, the authors report that one of the researchers, Harrie, found it difficult to balance her enteric feeding regime with the anticipated amount of walking, which totaled more than 200 miles between the research team over the course of the interviews. Further, Harrie, reflects on how a participant gave her a bottle of water, which she refers to as an act of care stemming from a “mutual disability solidarity” (29, p. 15). Through the framing of “care-full encounters”, the authors highlight the important role of reciprocity, solidarity and mutual understanding.

Promoting thinking beyond the participant-research division, Komorowska-Mach, Zieliński, and Wojdat (3) centre the experiences of academic co-author Konrad, writing about co-creating ethical relationships through care and rapport. The authors write specifically about post-laryngectomy (larynx removal) communication. Konrad, like participants in his study, is also a person living without a larynx, and experiences disturbed ability to produce speech, and other anatomical changes related to breathing and eating. Konrad firmly rejects the label “vulnerable” and claims that many participants would not feel respected if they were considered in that way. The authors note that from the methodological point of view, the project underwent important changes, yet attribute this to findings from initial data collected as opposed to Konrad's insight. The authors do however tell that, through Konrad's insight into this supposedly vulnerable population, their thinking shifted from a somewhat stereotypical treatment of both the research group and the researcher-participant relationship, to an emphasis on building relationships founded on mutual care and rapport. The authors found that this revised perspective fostered ethical collaboration that was beneficial for all parties involved. Whilst the papers discussed here center the researcher's injury, illness or disability, they do not reflect specifically on the pain needs of the researcher. It is this gap that the proposed study aims to fill. This paper now turns to outline the proposed study design.

## 3 The proposed study design

Most studies focused on IBD and school have adopted a quantitative methodology, using tools such as surveys to determine school attendance rates (30) and academic performance (31, 32). When research has adopted a qualitative methodology, this has typically been via traditional research methods such as interviews [e.g., (33) who undertakes individual interviews to explore the school experiences of children with IBD]. Gordon's (33) study highlights the value in seeking the first-hand perspectives of children with IBD about their school experiences. An exception to this is a study exploring friendships and IBD (34, 35). In this study, face-to-face interviews, friendship maps, and photographs were used within a participatory framework to explore whether young people tell or do not tell friends about their IBD, and how friendships form or fail. The authors reflect on how they developed a sensitively and carefully prepared topic guide with guidance from young people, the literature, researcher experience, and in collaboration with experts from clinical practice, owing to the sensitivity of the topic.

Recognizing the relative lack of qualitative research undertaken into the school experiences of children and young people with IBD, the proposed project will employ a qualitative methodology. Specifically, this methodology will be creative, cathartic and care-full. Creative cathartic methodologies is a term used by Madge (36) in her study of living through, with and on from breast cancer. Madge (36, p. 207) argues that employing a creative cathartic methodology can prompt an “opening into learning” that provokes emotional enquiries about what it means to be taught by the experience of others. I extend this term to include the notion of “care-full” research. Care-full qualitative research is a term used by Budworth (37), drawing on the feminist ethics of care literature, to promote a flexible response to the complex lives of research participants with chronic illness, also reducing ableist and exclusionary research encounters. Creative, cathartic and care-full methods are of value as they allow for responsiveness to the embodied and fluctuating nature of participants' chronic illnesses (37), which may be shaped, for instance, by flare-ups and periods of remission of acute pain.

Flexible research approaches have been utilized by Crip^2^ Theorists and Critical Disability Scholars (37). Such methods prioritize the “comfortabilities and capacities” of chronically ill participants (37, p. 1). For instance, children with IBD may be concerned about locating the nearest toilet if research is conducted at an unfamiliar venue. Further, if face-to-face workshops were held, for instance, children who are on biologic or steroid treatment for their IBD and are immuno-compromized may be put at unnecessary risk of infection. However, whilst not acknowledged in any research I have come across, these things matter for the researcher too – for instance, I too would have concerns about where to locate the nearest toilet if researching at a venue I was unfamiliar with, and I too am on biologic treatment and therefore at greater risk of infection.

Sander (14) raises a valuable point that while traditional qualitative methodologies aim to minimize the distance between the researcher and participants, they presume that they belong to two different worlds. In my research, the participants and I exist in some ways in the same world, living with the same chronic illness and managing similar symptoms, including those related to pain. Many researchers [e.g., (39–41)] have posed methodological considerations when researching illness and injury, including those characterized by pain. Literature has focused on how to design studies to accommodate the participant's pain (42) and to reduce the burden of participation for chronically ill participants (43). Informed by my own personal lived experience, supported by academic literature, and also shaped by feedback from Public and Patient Involvement and Engagement (PPIE) feedback from children and young people with a bowel condition (*n* = 4) and parents of a child or young person with a bowel condition (*n* = 4), the final informed study design for “IBD, School and Me” is detailed below. It is important to note that data collection has not yet taken place, and thus there may be new learning, accommodations and adaptations to these methods that would be useful to reflect on in the future.

### 3.1 Virtual interviews

Remote methods cover a broad range of methods and include videoconferencing interviews, referred to herein as virtual interviews. Videoconferencing as a research platform for conducting interviews has been praised for its flexibility, convenience and authenticity (44). Whilst reported limitations of remote methods include a failure to capture nonverbal cues of the wider body (beyond facial expression), and a greater risk of participant no ‘shows’ [see Khan and MacEachen (45)], I argue that the benefits outweigh the limitations, particularly when considering that remote methods have been recognized as supportive of what is referred to as “Crip Time” (46, p. 27). Crip Time acknowledges the need for extra time when living as a disabled person, whilst also highlighting the importance of flexible time to meet the needs of the body, as opposed to forcing the body to fit normative clocks and practices (47). This connects to Miserandino's (48) Spoon Theory which promotes the limited number of spoons (energy) available each day to chronically ill people, which are used when completing everyday mundane tasks, such as taking a shower, getting dressed, preparing food etc. Considering the limited number of spoons a chronically ill person may have, it would be unfair to expect them to participate in research which may deplete these spoons needlessly. For instance, it would not be acceptable to hold an interview in a city center venue which requires a participant, or indeed the researcher, to take multiple modes of transport to access a venue, when the same interview could be held virtually and attended from the comfort of their home.

In the “IBD, School and Me” study, virtual interviews will be undertaken via Microsoft Teams with children and young people with IBD, accompanied by a parent/carer for those under the age of sixteen. Virtual interviews prioritize, as discussed above, the “comfortabilities and capacities” (37:1) of both the participant and the researcher the IBD. Many virtual interviews have employed additional approaches or techniques within the interviews to effectively gather data with the identified participant group, for instance Carter et al. (49) used photo-elicitation within virtual interviews with young adults with chronic pain. Within the virtual interviews in the proposed study, a Persona Doll approach will be utilised to explore the everyday school lives of the younger children with IBD^3^. Ground rules will be outlined at the beginning of the interview to note that both participants and the researcher will have the option to pause the interview at any time for a break. Discussing the potential use of virtual interviews with the PPIE group of children and young people with a bowel condition and parents of a child or young person with a bowel condition helped to shape the use of this method. For instance, suggestions included allowing use of the chat function to type either all or some responses, including those a participant may feel more embarrassed to share verbally, and permitting participants to turn the camera off, again either for the full duration of the interview, or for responses a participant may feel more embarrassed to share.

### 3.2 Participant diaries

One burgeoning area of research interest, partly stemming from the Covid-19 pandemic and restrictions put in place to face-to-face research, concerns asynchronous methods, including internet meditated focus groups (51, 52) and email interviews (53). However, not all asynchronous research approaches have to be virtual/electronic. Asynchronous research is simply an approach in which the respondent records their response on their own time, within a specified time frame. Benefits of this approach for participants with pain include having no pressure to participate in a research study at a pre-determined time and date, when their pain may not allow for this on the day itself. Reflecting on the use of asynchronous focus groups for researching culturally sensitive issues, MacNamara et al. (51) recognize that asynchronous focus groups allow participants to provide responses at a time that is conducive to their own needs. They conclude that this research approach provides participants with a safe space, more time, and to contribute at their own pace to a research study. These features – safety, time and pace – are important features of a research study not only for participants, but also for a researcher living with pain.

In the IBD, School and Me study, participant diaries will be used as an asynchronous research tool, recognizing its value in the terms MacNamara et al. (51) has discussed above. Feedback from the PPIE group of children and young people with a bowel condition and parents of a child or young person with a bowel condition provided insight into the familiarity of keeping a diary for many children and young people with IBD. For instance food diaries, tracking symptoms and recording of possible medication side effects. The participant diary will allow for what I term nocturnal research participation. Many people with chronic pain experience pain which interrupts their sleep. “Painsomnia” is a term created by patients to describe difficulty falling or staying asleep due to chronic pain. A participant diary would enable a participant who could not sleep due to their pain, or was awoken due to their pain to participate in research in this time, should they wish and feel able to. This is in contrast to some other research which tends to take place during the researcher's own working day (mostly 9am–5pm). It should be noted, however, that since the Covid-19 pandemic, academics are increasingly working outside of the traditional “9–5” work day (54), with many Higher Education institutions placing emphasis on flexible working and compressed hours working, and therefore this 9–5 model of research participation is arguably no longer truly reflective of academic working practices, and research practices should too be flexible.

Solicited diaries have been used in previous research to access everyday experiences (55, 56). The usefulness of diaries as a methodological tool is attributed to their ability to facilitate access to emotional spaces and situations (55) and for eliciting the “felt, touched and embodied constitution of knowledge” (57, p. 501). Beneficially, the solicited diary is a portable method (58), and therefore can engage with a variety of spaces. Whilst much literature incorporating the use of diaries as a research method have reflected on their benefits to the research study, as noted above, there has been scarce reflections on the benefits of this approach for the researcher. For instance, as a researcher living with pain, the participant diary is a relatively “hands-off” research method, in the sense that beyond handing the diary out and collecting it in at the end of the data collection period, and some “checking in” communication throughout the duration of completing the diary (which can be done via telephone or email communication), there are no further physical demands on the researcher's body, giving it time to rest and recuperate. This makes it an appropriate method for a researcher living with pain, in contrast, for instance to travelling to scheduled in-person interviews or coordinating focus groups.

## 4 Conclusion: towards painless and productive research relationships

This paper has provided insight into pain and relationships from the perspective of a researcher with chronic pain designing a research study for participants with chronic pain. In a move towards shared vulnerability, I have explored the decisions I made surrounding research design, ensuring this was creative, cathartic and care-full. I have explained how the chosen methods of data collection (virtual interviews and participant diaries) accommodate both my own pain, and the anticipated pain experienced by the research participants, to foster the most productive and inclusive research relationships. These methods were recognized as flexible, being able to take place in a safe space (likely in the researcher and participants own homes or another location identified as safe), whilst the diary method to be undertaken at the participant's own pace, which is undoubtedly beneficial for a participant who is living with pain. However, these methods were also recognized as being appropriate for a researcher living with pain – for instance, the virtual interviews meant that there was no over-exertion caused by travelling to venues when in pain. Further benefits for myself living with multiple chronic illnesses include the removal of anxiety that comes with searching for toilets in a public space, or no additional threat of illness to an immunocompromised body. The diary method was recognized as a “hands-off” method, requiring little physical intervention from the researcher, therefore allowing for rest and recuperation when living with pain.

However, more than this, through this paper I have reflected on how there is, understandably, a centering of participant's needs during the design of a research study, with institutional ethics forms and the respective research ethics committees concerned about how a proposed study is appropriate for the prospective participants. Whilst not denying the importance of this, this paper has argued that the researcher's needs must be center-stage too. Whilst there is some existing evidence of this in relation to keeping researchers safe (e.g., questions around lone working), and concerns around minimizing distress to the researcher when researching a sensitive subject, the physical impact of undertaking research for the researcher and the demands on the researcher's body have not been given due attention. This paper recommends that institutional ethics committees and protocols, guidance and frameworks for research ethics issued by funders and other field and disciplinary organizations, need to widen their focus to give due attention to ethical issues related to study design from the researcher's perspective, as well as that of the participant, believing that this will help to foster the most productive and inclusive researcher-participant relationships. For instance, including prompts in ethics applications to justify if the proposed methods have been chosen to allow for reasonable adjustments for the researcher, for instance. As such, this article offers an important shift in thinking which will allow research to be undertaken in ways that are mutually supportive of participants and researchers. Indeed, attending to the researcher's needs may also be relevant for those managing other health and wider conditions including neurodiversity, mental illness, and pregnancy. I therefore end this paper with a call for other researchers to center themselves, alongside their participants, in study design and research ethics in a move towards shared vulnerability.

## Data Availability

The original contributions presented in the study are included in the article/Supplementary Material, further inquiries can be directed to the corresponding author.

## Footnotes

^1^For an exception, I refer the reader to Bashir (11) who discusses researcher encounters that unfold in such a way that the researcher becomes the “vulnerable”, fearful of being on unfamiliar territory, anxious about the unpredictability of participants; and feelings of being powerless to help.

^2^The term “cripple” has historically been used to arouse pity or disdain for a disabled figure. Crip is a reclaimed term which has been used as a marker of proud identification, resisting attempts to diminish or devalue disability or disabled people (38).

^3^Persona Dolls are fabric dolls that are used as part of a specific approach (the Persona Doll approach) to encourage inclusion and to challenge inequality and discrimination. I am trained in the Persona Doll approach by Persona Doll UK [see Wilkinson and Wilkinson (50)].
